# *In-operando* high-speed tomography of lithium-ion batteries during thermal runaway

**DOI:** 10.1038/ncomms7924

**Published:** 2015-04-28

**Authors:** Donal P. Finegan, Mario Scheel, James B. Robinson, Bernhard Tjaden, Ian Hunt, Thomas J. Mason, Jason Millichamp, Marco Di Michiel, Gregory J. Offer, Gareth Hinds, Dan J.L. Brett, Paul R. Shearing

**Affiliations:** 1 Electrochemical Innovation Lab, Department of Chemical Engineering, University College London, Torrington Place, London WC1E 7JE, UK; 2 ESRF, The European Synchrotron, 71 Rue des Martyrs, 38000 Grenoble, France; 3 Synchrotron Soleil, L'Orme des Merisiers, 91190 Saint-Aubin, France; 4 Department of Mechanical Engineering, Imperial College London, South Kensington Campus, London SW7 2AZ, UK; 5 National Physical Laboratory, Hampton Road, Teddington, Middlesex TW11 0LW, UK

## Abstract

Prevention and mitigation of thermal runaway presents one of the greatest challenges for the safe operation of lithium-ion batteries. Here, we demonstrate for the first time the application of high-speed synchrotron X-ray computed tomography and radiography, in conjunction with thermal imaging, to track the evolution of internal structural damage and thermal behaviour during initiation and propagation of thermal runaway in lithium-ion batteries. This diagnostic approach is applied to commercial lithium-ion batteries (LG 18650 NMC cells), yielding insights into key degradation modes including gas-induced delamination, electrode layer collapse and propagation of structural degradation. It is envisaged that the use of these techniques will lead to major improvements in the design of Li-ion batteries and their safety features.

## Abstract (web summary)

It is important to understand the mechanisms of thermally induced battery degradation and any safety hazards. Here, the authors use high-speed synchrotron radiation X-ray computed tomography to shed light on the structural and thermal dynamics associated with thermal runaway and failure of commercial Li-ion batteries.

## Graphical abstract

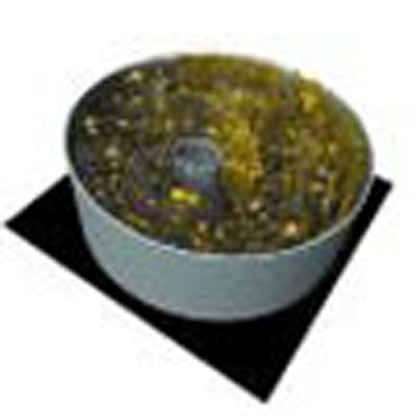

Lithium-ion (Li-ion) batteries are recognized as an integral technology in the process of achieving a clean and sustainable energy future^1^,^2^. The safety of Li-ion batteries is of utmost importance, in particular as the increasing deployment of electric and hybrid electric vehicles requires high-energy density batteries capable of operating under a wide range of demanding conditions, including elevated temperatures and high charge/discharge rates^3^,^4^. Although the probability of failure is relatively low, batteries can and do fail^5^, sometimes catastrophically; several well-publicised incidents have highlighted the need for enhanced battery safety^6^,^7^.

The thermal response of a cell is one of the most important characteristics to understand when assessing the safety of a cell design. Undesirable increases in temperature can occur within the cell as a result of electrical or mechanical abuse, or due to the presence of an external heat source, for example, failure of a neighbouring cell. Depending on the cell design there are a range of critical temperatures, which, when reached, will result in exothermic breakdown of its constituents. Once the rate of heat generation exceeds the rate of heat dissipation into the environment, the temperature of the cell starts to rise; thereafter, a sequence of detrimental events propagates in a process known as thermal runaway.

A temperature increase within the cell can trigger thermally induced degradation; for example, between 90 °C and 120 °C heat generation and gas evolution at the negative electrode occurs due to breakdown of the solid electrolyte interphase (SEI) and reaction between the lithiated carbon and the electrolyte^8^,^9^. Continuous exothermic decomposition and reformation of the SEI layer is believed to occur up to the highly exothermic breakdown of the graphite phase (*ca*. 220 °C)^10^. Common separator materials such as those based on polyethylene and polypropylene start to melt above *ca*. 130 °C, exposing the battery to the risk of local short circuiting^10^,^11^. Alkyl carbonate electrolytes start to break down at *ca*. 15 °C –200 °C^12^,^13^. Positive electrode materials such as LiNi_1/3_Mn_1/3_Co_1/3_O_2_ (NMC) and LiFePO_4_, begin to exothermically decompose at *ca*. 220 °C and *ca*. 300 °C, respectively, releasing oxygen^10^. However, the amount of oxygen evolved from degradation of cathode materials such as NMC is limited and further supply of oxygen from the surroundings is required for complete combustion of cell contents to occur^12^. Rapid exothermic reactions between electrolyte and lithiated graphite along with the rapid breakdown of lithiated graphite-negative electrode materials occur in the range *ca*. 200–260 °C 9,10,14. Without external intervention, this process will continue until complete depletion of reactants and frequently results in catastrophic failures of commercial cells such as fires and explosions, often with the release of flammable and toxic gases^15^,^16^,^17^. Commercial battery designs incorporate a number of safety mechanisms, such as pressure relief vents and positive temperature coefficient (PTC) devices. The function of a PTC device is to rapidly increase the resistance of the cell at high temperatures, to reduce the current and heat generation from the current in an attempt to prevent the onset or mitigate the effects of thermal runaway^3^,^18^, some of which leave the battery unusable after being triggered.

Although there have been studies on compositional and structural evolution of electrode materials at elevated temperatures^19^,^20^, there is limited understanding of the change in the internal architecture of commercial cells leading up to and during thermal runaway and failure. For example, gas evolution during thermal runaway is known to distort the structure of prismatic cells, causing delamination of electrode and current-collecting layers^21^. Internal short circuits may develop as a result of the displacement or distortion of the battery structure, in particular in pouch cells where gas-evolution-induced distortions are unconstrained. Improved understanding of the internal structural dynamics during failure and thermal runaway of commercial cells should help cell developers to design effectively against the initiation and propagation of failure.

X-ray computed tomography (CT) is a non-destructive tool used to acquire high-spatial resolution three-dimensional (3D) images of materials and devices, allowing researchers to visually inspect and quantitatively analyse material structural properties^22^,^23^,^24^. X-ray CT has previously been used to explore the microstructural properties of electrode materials in Li-ion batteries^25^,^26^,^27^,^28^,^29^ and has been shown to be an effective tool for diagnosing battery failure mechanisms *post-mortem*. Consecutive 3D images during *in-situ* analyses can be used to quantify microstructural evolution processes, facilitating identification of potential failure and degradation mechanisms^30^,^31^. However, to date, this has all been conducted at the electrode-length scale, during normal operation, not accounting for the dramatic changes to cell architecture that occur during catastrophic failure.

In this work, we use high-speed synchrotron radiation X-ray CT, combined with thermal imaging, to achieve unparalleled insight into the structural and thermal dynamics associated with thermal runaway and failure of commercial 18650 Li-ion batteries (2.2 Ah and 2.6 Ah 18650 LG NMC cells) during external thermal abuse. High-speed tomography at 1.25 and 2.5 Hz is performed during thermal runaway in the ID15A beamline at The European Synchrotron Radiation Facility (ESRF), which is renowned for its high-frequency imaging capability^32^. Through 3D reconstructions and two-dimensional (2D) radiographs captured at a rate of 1,250 frames per second (fps), we track for the first time the progression of rapid internal structural deformation leading up to and during thermal runaway. This new approach allows us to observe the effects of gas pocket formation, venting and elevated temperature on the architecture of internal spiral-wound layers of commercial Li-ion batteries and to evaluate the influence of engineering design on battery safety and performance.

## Results

### *In-operando* X-ray imaging

**Figure 1:**
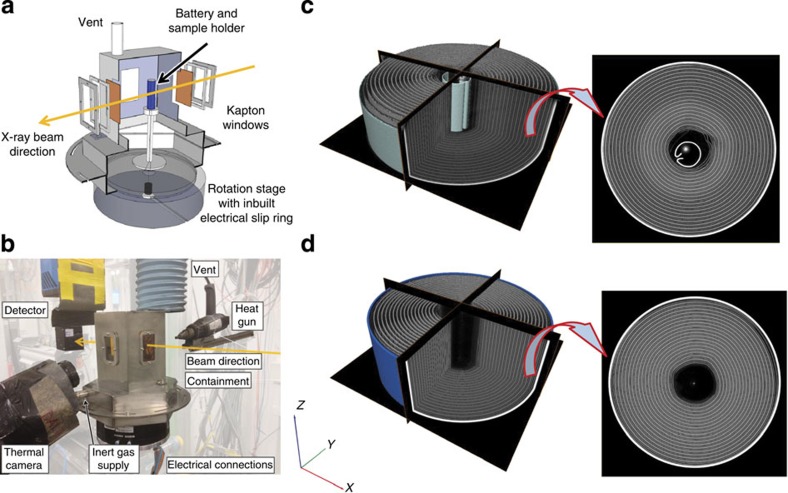
Schematic illustration of experimental setup and 3D reconstructions of battery cells. (**a**) Cut-away of battery containment design attached to the rotation stage for *in-operando* X-ray CT; (**b**) arrangement of apparatus for X-ray CT thermal runaway experiments; (**c**) 3D reconstruction with orthoslices in the *XY*, *YZ* and *XZ* planes of the 2.6 Ah battery (Cell 1) with isolated *XY* slice; (**d**) 3D reconstruction with orthoslices in the *XY*, YZ and *XZ* planes of the 2.2 Ah battery (Cell 2) with isolated *XY* slice.

Real-time tomography studies of rapid battery failure mechanisms were performed in ESRF's ID15A beamline using the setup shown in Fig. 1a,b and described in the Methods section. Two commercial LG 18650 NMC batteries (2.6 Ah and 2.2 Ah)^33^,^34^ in fully charged states (4.2 V as shown in Supplementary Fig. 1) were subjected to elevated temperatures (>250 °C) by the application of a heat gun. For simplicity, the 2.6 Ah and 2.2 Ah cells will henceforth be referred to as Cell 1 and Cell 2, respectively. Cell 1 contains an internal cylindrical structural support with inherent safety advantages^35^,^36^,^37^ (Fig. 1c) and was imaged at 1.25 Hz, whereas Cell 2 without the internal support (Fig. 1d) was imaged at 2.5 Hz. The reconstructed tomograms consist of grey-scale image stacks in which the most highly X-ray-attenuating materials are displayed as white and the least attenuating as black; steel and copper are highly attenuating materials and are displayed as white in the tomography and radiography images. The nickel manganese cobalt oxide (NMC) electrode material is displayed as bright grey. Aluminium, graphite and the separator materials exhibit low attenuation of the X-ray beam and are difficult to distinguish; however, their position can be derived relative to the copper and NMC phase.

### Temperature measurement

**Figure 2:**
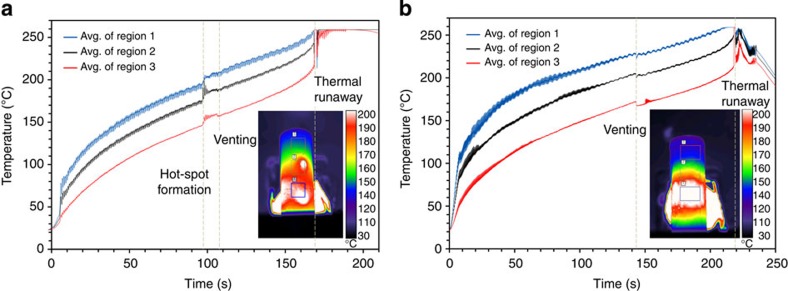
Temperature measurement. (**a**) Mean surface temperature profiles of the three regions (shown in the thermal image) on Cell 1 during the thermal abuse test showing thermal runaway after 168 s. The hotspots shown in the thermal image occurred on the surface of the shell after 97 s in Supplementary Movie 1. As judged from the thermal image, the spot size of the heat gun on the surface of the cells was *ca*. 10 mm in diameter. (**b**) Mean surface temperature profiles of the three regions on Cell 2 during the thermal abuse test showing thermal runaway after 217 s. The thermal image was extracted from Supplementary Movie 2 after 80 s of heating.

The batteries were continuously rotated during the fast tomography experiments, which resulted in a circumferentially uniform heat distribution around the cylindrical shell. A vertical temperature gradient was observed due to alignment of the heat gun with the lower part of the cell. The evolution of the mean temperature of surface regions at three different heights is shown for each cell in Fig. 2. Real-time thermal imaging movies of the cells during testing are provided as Supplementary Material, Supplementary Movie 1 (Cell 1) and Supplementary Movie 2 (Cell 2).

The surface temperature of both cells rose rapidly for the first 10 s of the test and then continued to increase at a quasi-steady rate. Thermal runaway of Cell 1 occurred after 168 s (Fig. 2a) when its shell temperature reached *ca*. 230 °C; thereafter, the temperature of the battery exceeded the temperature range of the thermal camera (up to 260 °C). During thermal runaway, the shell and cap of Cell 1 remained intact, allowing the exothermic reactions to run to completion, and the rapid pressure rise inside the shell caused the molten contents to eject through the vent in the form of a jet of fluid (Supplementary Movie 1). In the seconds leading up to thermal runaway, a jet of hot gas, consisting of gaseous products from breakdown reactions, was observed emanating from the vent of Cell 1. This was followed by liquid, believed to consist of molten separator material, bubbling on the cap of the battery.

Thermal runaway of Cell 2 occurred after 217 s (Fig. 2b and Supplementary Movie 2). Venting of both cells is evident in the thermographs as a slight cooling due to the Joule–Thompson effect^16^, as observed after 105 s in Fig. 2a and 145 s in Fig. 2b. Venting at an earlier stage might expose the internal decomposition reactions to oxygen and moisture in the air, accelerating the occurrence of thermal runaway. The rapid pressure rise inside Cell 2 during thermal runaway caused the entire cap of the cell to detach and the mostly unreacted cell contents to eject from the casing before the exothermic reactions could run to completion. Consequently, the internal temperature of Cell 2 is not expected to have reached that of Cell 1, which remained largely intact during failure.

The thermal image in Fig. 2a shows the formation of two hotspots on the surface of the Cell 1 casing, which is evidenced by the sudden rise in temperature after 97 s. These hotspots are believed to originate from internal short circuits. The sudden appearance and transient nature of the hotspots suggest a mechanism involving a rapid but short-lived release of energy (Joule heating or exothermic reaction), which would be consistent with a severe short circuit, that is, one that results in a rapid local temperature rise with the greatest risk of initiating thermal runaway. Such short circuits are usually associated with the contact between highly conducting layers such as that between the two current collector foils or between the aluminium current collector and the graphite electrode, which could occur as a result of dendrite growth, impurities/shrapnel in the cell or internal structural collapse.

At temperatures above 130 °C the most probable internal short circuit is expected to occur between the cathode and the anode, as the separator layer begins to melt and lose its mechanical integrity. However, short circuits involving the cathode (NMC) and any other components are limited by the relatively poor conductivity of the cathode material and are not expected to exhibit local, rapid and short-lived temperature rises^38^,^39^. The rapid temperature rise observed over such a short time span is indicative of a short circuit between two highly conducting layers allowing high current flow: either between the aluminium current collector and copper current collector (Al–Cu) or between the aluminium current collector and the anode (Al–anode)^38^,^39^. Given the characteristics of the hotspots and their proximity in time to venting, it is inferred that they were either caused by gas-induced structural collapse inside the cell or by the aluminium current collector coming into contact with the negative shell casing as a result of the separator material melting or contracting at elevated temperatures. Internal short-circuiting is a major safety concern and cannot be prevented by mechanical or electrical safety devices^40^. This highlights the potential of thermal imaging both as a diagnostic tool, which could enhance thermal analyses during abuse testing, identifying short-circuiting resistances via dynamic thermal events and as an effective method for thermal model validation^41^.

### High-frequency tomography

Tomograms were captured at a rate of 1.25 and 2.5 Hz for Cell 1 and Cell 2, respectively, allowing visual monitoring of the internal structural degradation in the seconds leading up to thermal runaway. Supplementary Movies 3 and 4 show slices from the evolving tomograms of Cell 1 and 2 during the seconds leading up to thermal runaway. The lower-frequency tomograms were reconstructed using a larger number of images taken at smaller angular increments, which gave significantly improved 3D images (see Methods for more details). Higher frequency with reduced image quality was used where a large degree of movement was anticipated within the cell.

**Figure 3:**
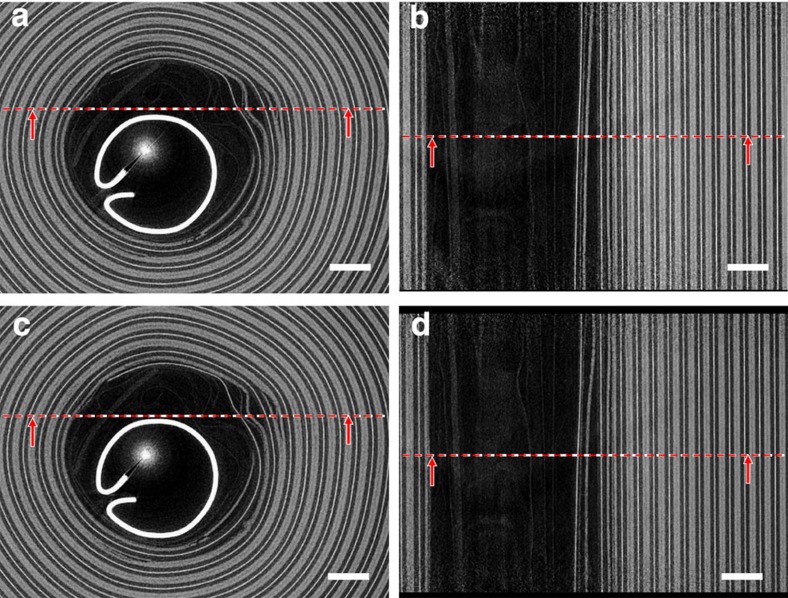
Grey-scale slices from 3D reconstructions in Supplementary Movie 3 during *in-operando* high-frequency tomography of Cell 1. (**a**) Enlarged grey-scale image of the *XY* plane 15 s before thermal runaway; (**b**) enlarged grey-scale image of the *YZ* plane 15 s before thermal runaway; (**c**) enlarged grey-scale image of the *XY* plane 1 s before thermal runaway; (**d**) enlarged grey-scale image of the *YZ* plane 1 s before thermal runaway. The dotted red lines indicate the through-plane slice with which the neighbouring image is associated. Scale bar, 1 mm.

At the core of Cell 1, there is a cylindrical support that aids in cell winding and provides mechanical support during manufacturing. Structural deformation around the inner layers of Cell 1 was observed leading up to thermal runaway (Fig. 3). The tightly wound electrode, current collector and separator layers remained mostly intact during thermal abuse; however, a small degree of delamination occurred around the inner region of the cell where the spiral-wound layers are least compressed. The separation of layers stemmed from the region between the positive and negative electrodes, that is, the separator/electrolyte region. The positive and negative electrodes in Fig. 3 are believed to have diverged due to melting of the separator layer and local gas generation from reactions such as SEI breakdown and formation, and electrolyte degradation. The extent of separation of the positive and negative electrode can be gauged from the side views in Fig. 3b, d, which show that the delamination continues vertically through the cell.

**Figure 4:**
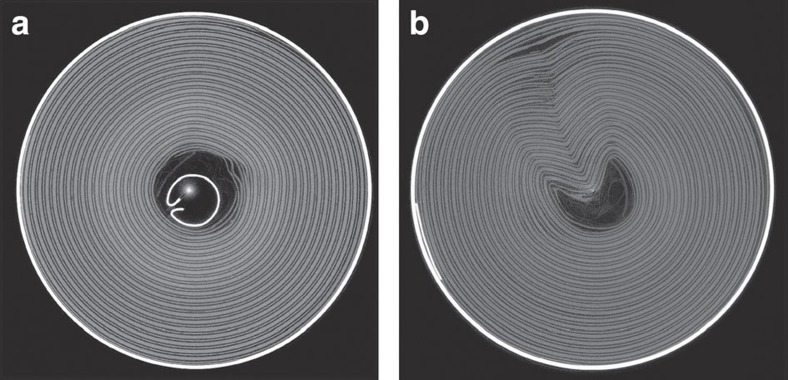
Post test images in the *XY* plane comparing the spiral layered structure. (**a**) Cell 1 with and (**b**) Cell 2 without an internal support. Cell 2, without the internal support, showed a severe structural collapse after venting.

The effect of the central cylindrical support is evident from the comparison of cell architecture after venting. Cell 1 has a tightly packed core with a steel cylindrical support, which helps prevent against internal collapse (Fig. 4a). No severe collapse was observed in Cell 1 as demonstrated in Fig. 4a. In contrast, no central cylindrical support is present in Cell 2, which showed a severe distortion of its architecture after venting (Fig. 4b). Before venting, thermally and electrochemically driven degradation reactions resulted in the formation of gas pockets between the electrode layers. It is believed that during pressure relief the gas pockets were rapidly channelled towards the vent via the path of least resistance, causing a sudden collapse in the structure of the spiral-wound layers. This collapse induced structural deformation including sharp bends in the layered materials, heightening the risk of severe internal short circuits and consequent thermal runaway, a risk that could have been reduced by incorporating a central support in Cell 2.

As seen in Supplementary Movie 3, little change in the internal macro-structure of Cell 1 was observed in the 15 s leading up to thermal runaway, with neither the NMC nor the graphite electrode showing any obvious signs of microstructural degradation. Both the NMC and graphite electrodes are expected to break down exothermically within a narrow temperature range (200 °C –250 °C) causing the cell contents to rapidly degrade^10^.

**Figure 5:**
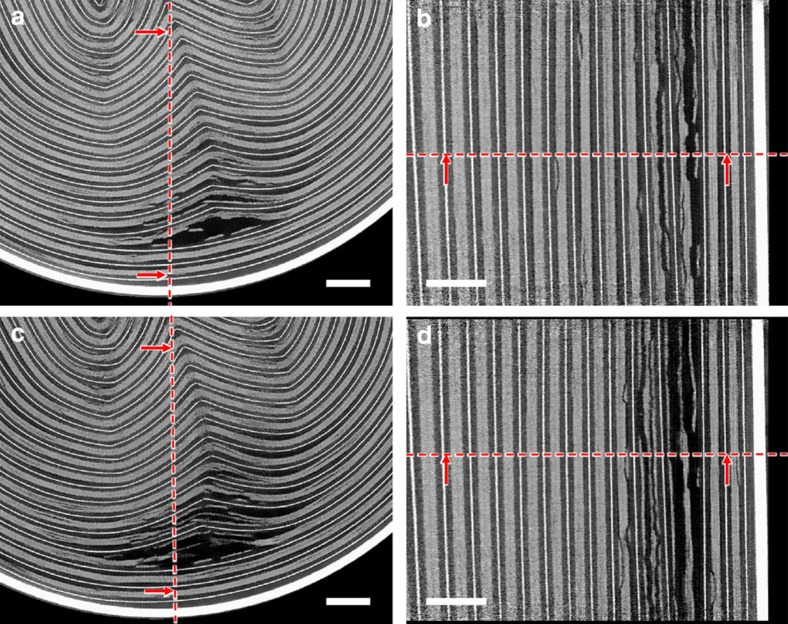
Grey-scale slices from 3D reconstructions from Supplementary Movie 4 during *in-operando* high-frequency tomography of Cell 2. (**a**) Enlarged grey-scale image of the *XY* plane 15 s before thermal runaway; (**b**) enlarged grey-scale image of the *YZ* plane 15 s before thermal runaway; (**c**) enlarged grey-scale image of the XY plane 1 s before thermal runaway; (**d**) enlarged grey-scale image of the *YZ* plane 1 s before thermal runaway. The dotted red lines indicate the through-plane slice with which the neighbouring image is associated. Scale bar, 1 mm.

In the 15 s leading up to thermal runaway, expansion of the collapsed region of Cell 2 and increasing divergence of the electrode layers was observed (Fig. 5 and Supplementary Movie 4). The progression of delamination and divergence originated from the interface between the positive and negative electrode, that is, the separator region in which gas generation from SEI and solvent decomposition is most prominent during thermal abuse^16^. Before thermal runaway, when the shell temperature is *ca*. 200 °C, broadening of the collapsed region was observed as gaseous products continued to flow towards the vent (Fig. 5a,c). After venting, the cell became open to the atmosphere and the widening collapsed region allowed penetration of the atmosphere deep into the cell, although only after initial venting when the flow rate had reduced sufficiently to allow diffusion back into the cell. For safety reasons a nitrogen atmosphere was used in this work, but in real-world situations this could accelerate the decomposition of the electrode materials and thermal runaway through the provision of oxygen. High-frequency tomography at 2.5 Hz could not capture the initiation and propagation of thermal runaway, as the substantial movement inside the cell during the catastrophic failure prevented reconstruction in 3D. However, the sequence of events during the initiation and propagation of thermal runaway was captured in the individual radiographs taken at 1,250 fps.

### High-speed radiography

**Figure 6:**
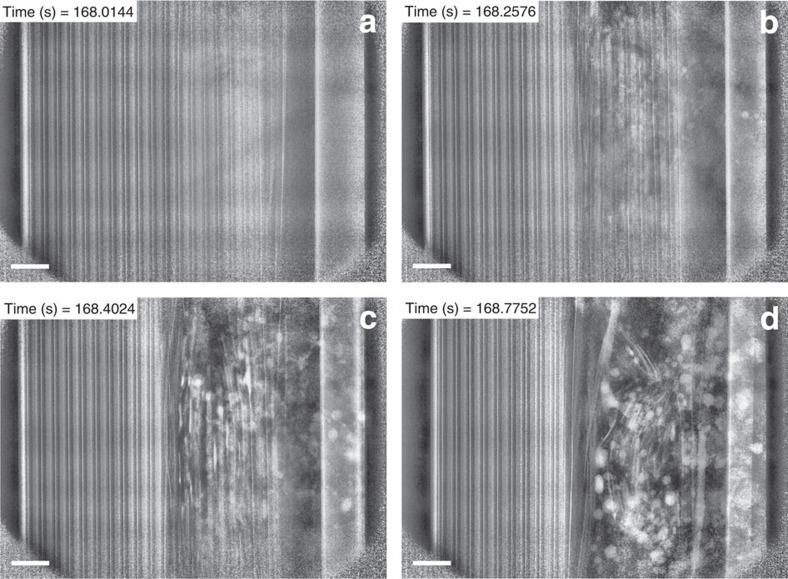
Radiographs from Supplementary Movie 5 showing the propagation of thermal runaway in Cell 1. (**a**) Radiograph of the *YZ* plane before thermal runaway; (**b**,**c**,**d**) sequential images showing the propagation of thermal runaway through the cell. The thermal runaway initiates at the inner layers where the maximum temperature is apparent and spreads radially outwards. The formation of copper globules can be observed as highly attenuating white blots in images **b**, **c** and **d**. Heating is applied from the right of the images but continuous rotation at 180° every 0.4 s maintains an even circumferential temperature distribution. Scale bar, 1 mm.

**Figure 7:**
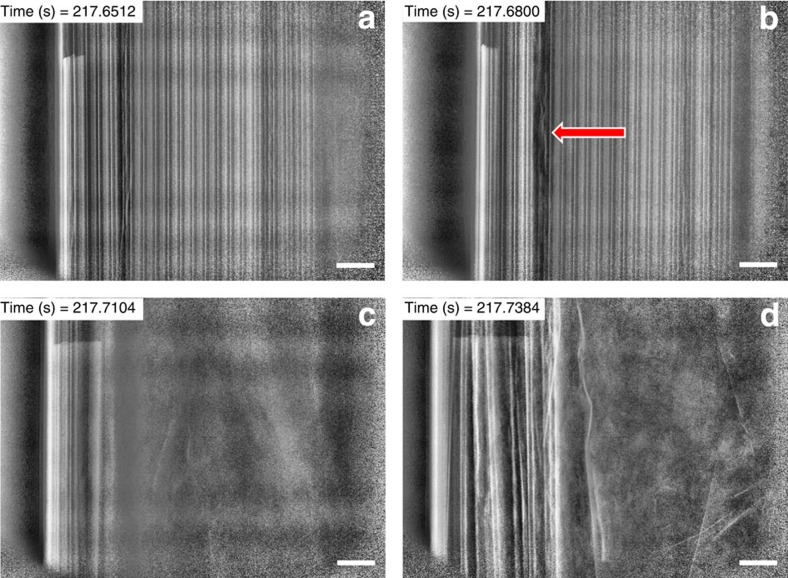
Radiographs from Supplementary Movie 6 showing the propagation of thermal runaway in Cell 2. (**a**) Radiograph of the *YZ* plane before thermal runaway; (**b**) radiograph during thermal runaway where the red arrow indicates the region in which structural breakdown is first observed; (**c**) radiograph during ejection of contents; (**d**) radiograph immediately after ejection of contents. The time-stamped radiographs show that the entire process of initiation and ejection lasted <0.1 s. Heating is applied from the right of the images, but continuous rotation at 180° every 0.2 s maintains an even circumferential temperature distribution. Scale bar, 1 mm.

By using high-speed radiography, we were able to observe events that were too rapid to be captured by high-speed tomography, thereby providing insight into the mechanism behind initiation and propagation of thermal runaway. The radiographs show a 2D integrated representation of the whole cell rather than individual focal plane slices such as those selected from the tomograms. Time-stamped radiographs taken at 1,250 fps and showing half the cell during thermal runaway were compiled into movies (Supplementary Movie 5 and Supplementary Movie 6). Still radiographs captured at various points during thermal runaway for Cell 1 and Cell 2 are presented in Fig. 6 and Fig. 7, respectively.

The initiation of thermal runaway in Cell 1 occurred above the field of view in Supplementary Movie 5 and in Fig. 6. As the NMC electrode exothermically decomposed, the combination of local gas generation and increasing temperature resulted in a rapid pressure rise within the cell. The consequent pressure difference between the region undergoing thermal runaway and the atmosphere appears to have fractured the electrode materials and directed the fragments towards the vent of the battery. Initially, the exothermic decomposition reactions proceeded between the copper current-collecting foil, which for as long as its mechanical integrity was maintained, channelled the electrode material fragments towards the top of the cell. The breakdown and ejection of electrode material spread radially outwards, providing evidence that the propagation of thermal runaway in Cell 1 occurred from the inner to the outer layers despite the heat being applied to the external surface. This may be due to the thermally insulating effect of the outer layers when exothermic reactions and internal short-circuiting occur, so that the temperature of the inner layers is first to significantly exceed the temperature of the shell.

Locally, the heating rate exceeds the heat dissipation rate. The resulting increase in temperature can be deduced from the observation of the copper current-collecting material melting and collecting into globules (highly attenuating white spots in Fig. 6) around the region of apparent electrode breakdown. This suggests the presence of a very large temperature gradient between the inside and the outside of the cell, as the inner region has reached the melting point of copper (1,085 °C), while the outer electrode layers remain intact. NMC and lithiated graphite are known to decompose at 300 °C^10^,^16^, implying a transient temperature gradient of over 700 °C within the cell during the propagation of thermal runaway. Following loss of mechanical integrity of the spiral layers, large portions of the spiral-wound materials were ejected together. Heat transfer from the exothermic reactions to the neighbouring layers was therefore interrupted as large sections of reacting material were ejected. The ejection of material to the atmosphere effectively dissipated the heat and mitigated the propagation of thermal runaway, although the effect on any surrounding cells would be exacerbated by the presence of air.

In contrast to Cell 1, thermal runaway was not observed to propagate throughout Cell 2 shortly after the onset of significant motion and degradation within the cell. As shown in Supplementary Movie 6, the bulk of the intact contents were ejected soon after signs of material degradation and thermal runaway initiation had been observed. The collapsed structure of Cell 2 (Fig. 4b) is seen in Supplementary Movie 6 as a black region near the left edge, which expands and contracts as the battery rotates.

The layered structure of Cell 2 remained intact up until 217.60 s (Supplementary Movie 6 and Fig. 7a), when the electrode layer indicated in Fig. 7b began to break down, followed by a sudden shift of bulk material towards the vent of the cell between 217.66 and 217.70 s in Supplementary Movie 6. Less than one tenth of a second later, the cell cap detached, the internal contents of the cell were ejected (Fig. 7c) and the propagation of thermal runaway was halted. The upward shift of intact material may have prevented the release of pressure during thermal runaway by blocking the vent. An uncontrolled failure occurred due to the hindered pressure relief and a rapid pressure rise inside the cell. Hence, the propagation of thermal runaway within the cell was hampered by the rapid dissipation of heat and the ejection of reacting contents to the atmosphere.

### *Post-mortem* tomography

**Figure 8:**
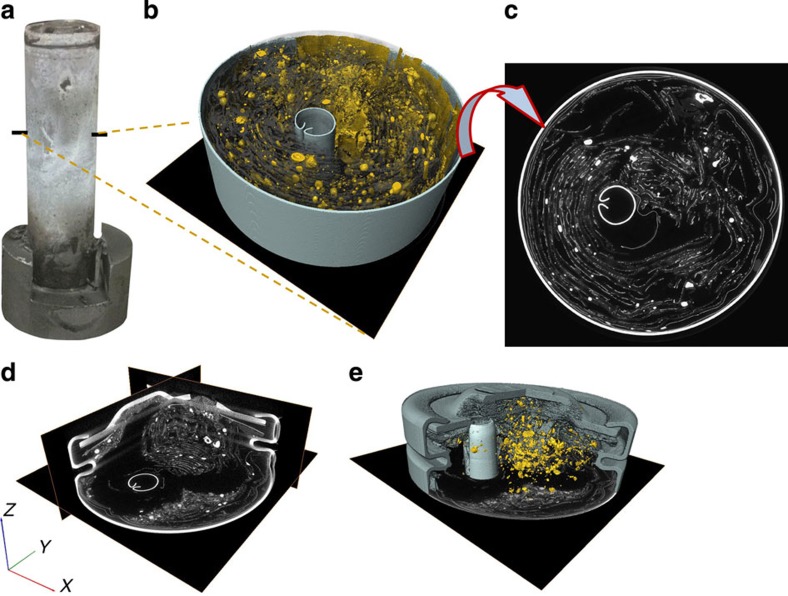
*Post-mortem* tomography of Cell 1 after thermal runaway. (**a**) External view of Cell 1 after thermal runaway where the black marks indicate the points at which the bottom slice of the corresponding tomogram begins; (**b**) 3D reconstruction showing isolated copper phase (yellow), other broken down material (semi-transparent dark grey), battery casing (teal) and central cylindrical support (teal); (**c**) grey-scale slice from the *XY* plane; (**d**) tomogram of the battery vent region showing grey-scale slices from the *XY*, *YZ* and *XZ* planes; (**e**) 3D reconstruction of the cap region showing the placement of the central cylindrical support near the vent.

*Post-mortem* tomography scans were taken to assess the internal aftermath of thermal runaway throughout both cells. The *post-mortem* 3D reconstructions of Cell 1 shown in Fig. 8 reveal a complete destruction of the original spiral-wound architecture. The copper phase is easily distinguished from the rest of the material in the reconstructed images due to its high attenuation and has been isolated from the rest of the contents in Fig. 8b. The presence of the large copper globules indicates that temperatures within the cell reached in excess of 1,085 °C (the melting point of copper).

Some intact remains of the copper current-collecting foil can be observed around the outer walls of the battery. The high concentration of large copper globules around the inner regions of the 3D reconstructions compared with the presence of intact current collector remains around the outside indicates an uneven temperature distribution within the cell during thermal runaway. The propagation of thermal runaway from the centre of the battery to the outer edges is also demonstrated by the radiographs in Supplementary Movie 5 and Fig. 6, and is consistent with the presence of a significant temperature gradient between the inner and outer layers. A large mass of fragmented material is seen to have gathered near the vent of Cell 1 and the central cylindrical support has aligned with one of the vent holes (Fig. 8e), which supports the hypothesis that the position of the cylindrical support hindered venting and ejection of material during thermal runaway.

**Figure 9:**
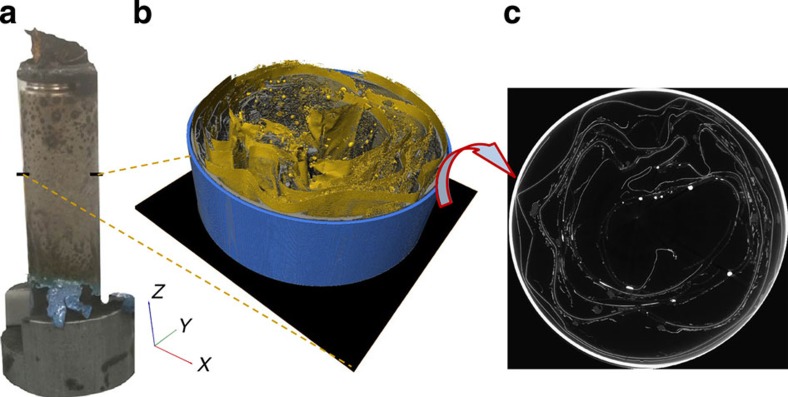
*Post-mortem* tomography of Cell 2 after thermal runaway. (**a**) External view of Cell 2 after thermal runaway showing the burst cap and protruding internal contents. The black marks indicate the points at which the bottom slice of the corresponding tomogram begins; (**b**) 3D reconstruction showing isolated copper phase (yellow), other broken down material (semi-transparent dark grey) and battery casing (blue) where the copper phase is mostly still intact; (**c**) grey-scale slice from the *XY* plane.

In contrast to Cell 1, the copper current-collecting foil in Cell 2 mostly remained in its original laminar form (Fig. 9). The ejection of the cell contents mitigated the propagation of thermal runaway and, consequently, the melting point of copper was not reached in the majority of the cell. As shown in Fig. 9b, small copper globules formed around the inner regions of the cell, which indicates that local temperatures reached above 1,085 °C but the propagation of exothermic reactions and heat transfer to the rest of the cell was interrupted by the sudden ejection of the cell contents. The radiographs of Cell 2 in Fig. 7 showed that the internal layered materials had not decomposed up until the point of rupture, suggesting that uncontrolled failure (detachment of the battery cap) is linked to the distance between the point of failure initiation and the vent. For example, initiation of thermal runaway near the bottom of the cell would result in the unreacted material above that point being forced towards the vent, thus increasing the likelihood of the vent region becoming clogged and the cap detaching from the cell. The possibility of the vent region clogging was previously highlighted in a study by Golubkov *et al.*^16^, where two venting events occurred during thermal abuse tests of 18650 NMC cells; one was due to the normal operation of the bursting plate and the second was attributed to relief after clogging of the vent. The 3D reconstruction in Fig. 9b and the slice in Fig. 9c show that the majority of the material remains are copper. In contrast to Cell 1, most of the softer electrode and separator materials, that is, the active materials that allow the propagation of thermal runaway, were ejected from the cell during the rupture. The *post-mortem* tomography in combination with the radiography results demonstrate that the bursting-cap failure mechanism may be advantageous in mitigating reaction propagation and heat generation inside such cells during thermal runaway.

## Discussion

*In-operando* high-speed tomography and radiography of Li-ion batteries during thermal runaway has proven to be an effective diagnostic tool for visualizing rapid failure mechanisms. The elucidation of internal structural features and dynamics has highlighted the effect of battery design on the safety and behaviour of cells during failure. This can only be achieved using non-invasive high-frequency imaging techniques. The evolution of gas pockets between the electrode layers was observed in the tomograms when the shell temperature exceeded 100 °C, resulting in electrode layer delamination. The formation of gas pockets reduces the electronically and ionically conducting interfacial area between the electrode layers and sustained gas generation leads to venting of the cell, a sudden release of pressure that can lead to collapse of the internal structure of the cell, depending on its design.

The magnitude of structural collapse observed in this study compromises the safety of the cell. A severe internal collapse increases the risk of internal short circuits, exposes decomposing material to oxygen and moisture in the air and consequently increases the risk of and accelerates thermal runaway at lower external temperatures. This risk may be avoided by including a tightly packed core or an internal structural support in the battery design^35^,^36^,^37^.

In addition, the location of thermal runaway initiation may have an effect on the failure mechanism of the battery; initiation of thermal runaway near the bottom of the cell may result in a mass ejection of intact contents above the point of failure, whereas initiation near the top of the cell would allow the propagation of thermal runaway to travel vertically down into the cell over time while contents are continuously ejected. The propulsion of the cell contents towards the vent may also render the function of the vent redundant if clogged, a phenomenon that will require further study. The failure mechanism of the battery can have a significant influence on the final temperature of the cell and on the rate and quantity of heat generated during failure. As Kim *et al.*^42^ highlighted, thermal runaway of a battery pack is likely to be induced by thermal runaway of a single failing cell, emphasizing the need to limit the heat transfer to adjacent cells. This can be achieved by installing heat barriers or intumescent layers between cells. As demonstrated in this study, mitigating heat generation and limiting the maximum temperature reached during thermal runaway by ejecting the degrading material could also contribute to safer modular designs.

High-speed radiography of Cell 1 (Supplementary Movie 5) provided striking images of the propagation of thermal runaway and evidence for the presence of an inhomogeneous temperature distribution within the cell, with the outer regions remaining intact while the inner regions lost their structural integrity as temperatures exceeded 1,085 °C. The relatively slow venting of Cell 1 helped contain the process of thermal runaway and allowed the decomposition reactions to run to completion, generating large amounts of heat, which emphasizes the importance of effective thermal management systems^43^. The early ejection of battery contents in the case of Cell 2 holds the advantage of preventing further heat generation and temperature increase, thus protecting neighbouring cells and the rest of the pack from the risk of thermal runaway. However, this should be balanced against the risk of heat generation on the external surface of neighbouring cells arising from oxidation of the ejected cell contents in air.

In summary, *in-operando* high-speed synchrotron radiation X-ray tomography and radiography, in combination with thermal imaging, have provided unprecedented insight into the structural and thermal dynamics leading up to and during thermal runaway and failure of Li-ion batteries during external thermal abuse. These discoveries enhance our understanding of the most dangerous failure mechanism of such devices and highlight the impact of engineering design on the performance and safety of 18650 cells. The containment design and arrangement of apparatus, including the electrical slip-ring described in this work, also facilitates X-ray imaging and thermal analysis of Li-ion batteries during electrical abuse, which would complement previous *in-situ* work^44^. However, the PTC in the commercial 18650 cells studied here prevents the high charge/discharge rates and extreme states of overcharge and undercharge required for catastrophic failure. A modified version of this set-up would also enable high-frequency imaging of thermal runaway induced via mechanical abuse such as nail penetrations and crush tests, highlighting the potential for this technique to uncover much more information across a wide range of abuse conditions.

The results shown in this study may also provide valuable information for multiphysics modelling of Li-ion battery failure, as structural distortion, breakdown and material ejection are not considered in current modelling strategies but are critical for accurate prediction of heat generation and dissipation. Dynamic visualization of structural degradation leading up to and during thermal runaway would support the development of multiphysics models of the effects of various abuse conditions and the onset of thermal runaway. These insights are expected to guide the development of safer and more reliable Li-ion battery designs.

## Methods

### High-frequency X-ray CT

The tomographic imaging was carried out in the ID15A beamline at the ESRF^32^, using a 76-keV monochromatic beam with a pixel resolution of 10.87 μm. Radiographs were recorded by the camera detector at a rate of 1,250 fps; tomograms were captured at a rate of 2.5 and 1.25 Hz, which comprised 500 and 1,000 projections, respectively. The tomograms with the lower number of projections were captured at a higher rate but significantly sacrificed image quality. Higher image quality and lower frequency are preferred where little movement within the batteries is expected.

The 2.5- and 1.25-Hz tomogram projections were taken over a 360° rotation of the cell at angular increments of 0.72° and 0.36°, respectively. The monochromatic beam had a field of view of 10.5 mm × 7.6 mm, which captured half of the cell in the radiographs. The transmission images were reconstructed into 7.6-mm-high 3D cylindrical sections of the cell using standard reconstruction methods^45^. The set-up presented in Fig. 1b provides the unique capability of real-time tomography, thermal and electrochemical analyses during electrical and thermal abuse tests, extending dynamic failure analyses of commercial cells to include internal structural features.

### *Operando* high-speed imaging

The imaging experiments were carried out using a 76-keV monochromatic beam with a field of view of 10.5 mm × 7.6 mm. The exposure time was set to 0.35 ms and the high-speed camera used for the imaging was a PCO Dimax (PCO AG, Germany), which achieved 1,250 fps. A region of interest of 960 × 700 pixels (horizontal × vertical) was used. Tomograms composed of 500 projections with a resolution of 10.87 μm were captured every 0.4 s over a 360° rotation at 150 r.p.m. Tomograms composed of 1,000 projections with a resolution of 10.87 μm were captured every 0.8 s over a 360° rotation at 75 r.p.m. To achieve the required tomogram frequencies, the images were stored on the PCO Dimax camera which, with 36 GB of on-board memory for fast intermediate storage, could store 17 s of recordings under these conditions. A post event triggering capability was implemented, whereby the detector recorded continuously until triggered, at which point it would store the previous 17 s of high-speed tomograms. During high-speed tomography, the sample was continuously rotated through 360°.

### Data processing

The transmission images were reconstructed in 3D using standard reconstruction methods^45^ and saved as 32 bit float files. The reconstructed tomograms were processed using Avizo Fire 7 (FEI VSG, France), where the 3D images were displayed as stacks of 2D image slices. An edge preserving non-local means filter^46^ was applied to the images, to remove noise, while preserving fine features and edge definition. Material phases were defined by grey-scale value using Avizo's segmentation editor and selectively displayed.

The radiography movies were developed using MATLAB R2013a. Flat field correction was applied to the raw radiographs to remove artefacts and the grey scale image contrast was enhanced using MATLAB's ‘adapthisteq' function for improved feature recognition. Images were converted to 16-bit unsigned integers before compiling into.avi movies.

The real-time thermal imaging movies and mean temperature versus time graphs were developed using FLIR's Altair software.

### Thermal abuse tests

A safe containment system (Fig. 1b) for thermal runaway of the selected commercial cells was designed to fit the ID15A rotation stage (ABR1000, Aerotech, USA). The containment system included a continuous inert (N_2_) gas flow to flush out any evolved gases and help prevent the propagation of fire, two X-ray transparent Kapton windows in the path of the beam, an infrared transparent sapphire window for thermal imaging and a fourth opening for inserting a heat gun (to simulate the external heating effects of battery pack failure). The system was not completely sealed and therefore the internal environment was not entirely inert; sufficient oxygen in air was still present to allow some limited ignition of the cell contents during thermal runaway. A uniform layer of heat-resistant black paint (with a calibrated emissivity^47^ of 0.96 over the range of 40–180 °C) was applied to each battery before testing. The batteries were tightly secured between a fastening screw and the wall of the sample holder, and were positioned so that the region of interest was in line with the beam. Heating was applied by a heat gun (HG 2310 LCD Electronic, Steinel, Germany) located next to the battery, which had a 9-mm reduction nozzle to focus the heat onto the battery surface, protecting neighbouring equipment and helping prevent circumferential heterogeneity in temperature. The reduction nozzle resulted in a spot size with *ca*. 10 mm diameter on the surface of the rotating batteries. The heat gun used a Duratherm heating element and precise control of the applied temperature was achieved by varying air flow rates and air temperature at 10 °C increments.

The temperature of the battery was recorded using a thermal camera (FLIR SC5000MB, FLIR Systems France, Croissy-Beaubourg, France), from the opposite side to the heat gun as shown in Fig. 1b. The thermal camera was calibrated for temperature ranges of 15 °C–250 °C and 250 °C–1,500 °C; the former temperature range was applied to ensure accurate temperature measurements over the expected temperature range^48^. The thermal camera had an extended wavelength detector, allowing detection of infra-red wavelengths between 2.5 and 7 μm. The noise equivalent temperature difference of the camera in the calibrated range was <20 mK. Images were recorded at a rate of 25 Hz and the temperature measurement accuracy, as specified by the manufacturer, was ±1 °C or ±1%.

Two commercial 18650 Li(NMC) batteries (LG 2.2 Ah NMC and LG 2.6 Ah NMC)^33^,^34^ were subjected to high temperatures (*ca*. 250 °C) using the heat gun, which was activated when the batteries began to rotate. The heat gun was set to a temperature of 480 °C at maximum airflow and the reduction nozzle of the heat gun was carefully placed 2 cm from the cells to avoid disparities in heating between tests. The continuous rotation helped to maintain a uniform circumferential temperature distribution around the batteries. Heating continued until thermal runaway occurred. The PCO Dimax camera stored 17 s at 1,250 fps under the conditions previously outlined. The post-event triggering for recording the images was activated *ca*. 2 s after the catastrophic outcome of thermal runaway (explosion or violent venting). This resulted in *ca*. 15 s of high-frequency tomograms being captured before the event and *ca*. 2 s of tomograms being captured after the event.

## Author contributions

D.P.F. and P.R.S. conceived and designed the experiments and setup. D.P.F., I.H. and G.O. carried out thermal and electrical abuse tests at Imperial College before experiments at the ESRF. T.J.M. and J.M. assisted with the mechanical design and manufacture of the battery containment. D.P.F., M.S., J.R., B.T., I.H., M.D.M. and P.R.S. performed the imaging and tomography experiments at the ESRF. M.S. and M.D.M. set up the beamline conditions and imaging strategies for high-frequency imaging. M.S. and D.P.F. carried out the image reconstructions. P.R.S., G.H. and D.J.L.B. provided constructive advice for preparing the results and writing the manuscript. D.P.F. performed the data analysis, interpreted the results and wrote the manuscript.

## Additional information

**How to cite this article**: Finegan, D. P. *et al.* *In-operando* high-speed tomography of lithium-ion batteries during thermal runaway. *Nat. Commun.* 6:6924 doi: 10.1038/ncomms7924 (2015).

## Supplementary Material

Supplementary Figure Supplementary Figure 1

Supplementary Movie 1 Thermal imaging. Real-time thermal imaging video of Cell 1 during thermal abuse.

Supplementary Movie 2 Thermal imaging. Real-time thermal imaging video of Cell 2 during thermal abuse.

Supplementary Movie 3 Structural evolution of the contents in Cell 1 during seconds leading up to thermal runaway. Individual slices are extracted from the 3D tomogram to highlight the structural dynamics however little change is observed in Cell 1 leading up to thermal runaway.

Supplementary Movie 4 Structural evolution of the contents in Cell 2 during seconds leading up to thermal runaway. Individual slices are extracted from the 3D tomogram to highlight the structural dynamics. Time-lapse tomogram slices show significant structural degradation around the collapsed region in the seconds leading up to thermal runaway.

Supplementary Movie 5 High speed radiography video showing half the YZ plane. The propagation of thermal runaway within Cell 1 is observed at 1250 fps. The thermal runaway initiates at the inner layers and spreads radially outwards. The formation of copper globules can be observed as highly attenuating white blots start to form around 168.26 s. Heating is applied from the right of the images but continuous rotation at 180 ° every 0.4 s maintains an even circumferential temperature distribution.

Supplementary Movie 6 High speed radiography video showing half the YZ plane. The propagation of thermal runaway within Cell 2 is observed at 1250 fps. The cell ruptures at around 217.69 s, ejecting its contents. Heating is applied from the right of the images but continuous rotation at 180 ° every 0.2 s maintains an even circumferential temperature distribution.
