# Supplementary material for: In-operando high-speed tomography of lithium-ion batteries during thermal runaway
Source: Nat Commun. 2015 Apr 28;6:6924. doi: 10.1038/ncomms7924 (PMC4423228; doi:10.1038/ncomms7924)
Supplement: Supplementary Figure — 1 [file ncomms7924-s1.pdf]

Supplementary Figures

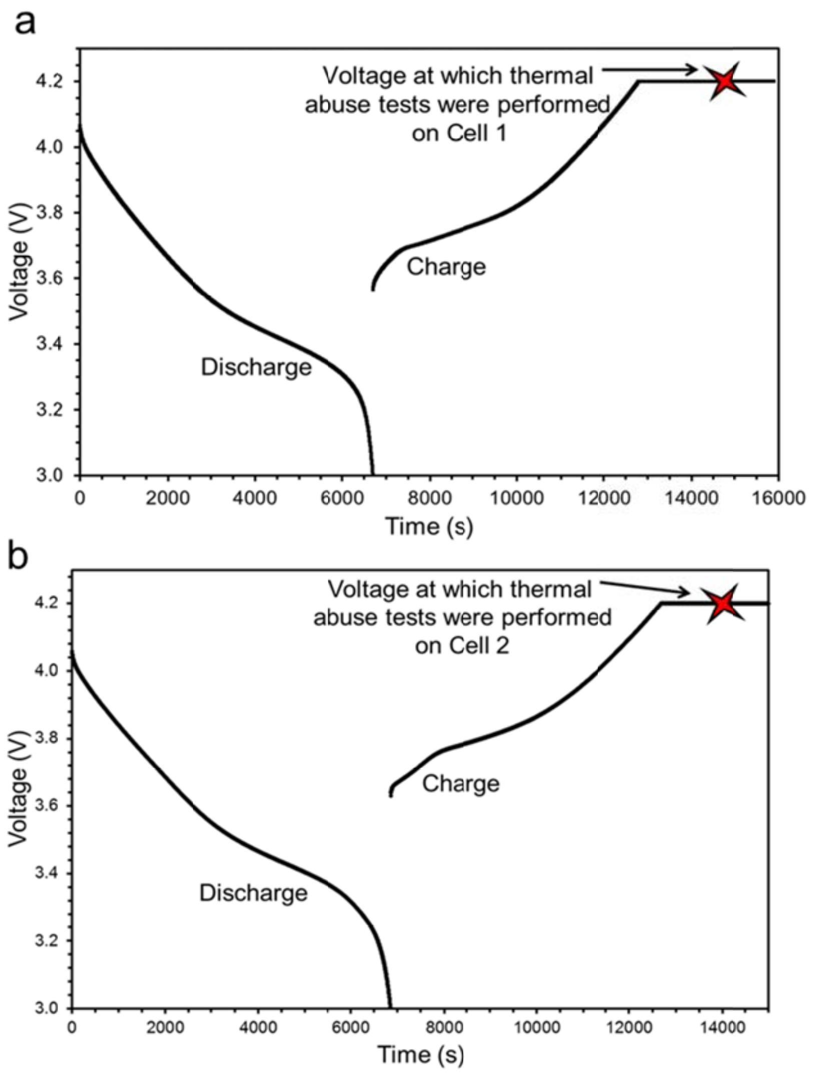

Supplementary Figure 1 | Charge and discharge curves at 0.5 C showing the state of charge at which both cells were tested. (a) Cell 1 (2.6 Ah) charged and discharged at 1.3 A and was tested under thermal abuse in a fully charged state of 4.2 V; **(b)** similarly Cell 2 (2.2 Ah) shown here to be charged and discharged at 1.1 A was tested under thermal abuse in a fully charged state of 4.2 V.
